# An Improvement of the Fire Detection and Classification Method Using YOLOv3 for Surveillance Systems

**DOI:** 10.3390/s21196519

**Published:** 2021-09-29

**Authors:** Akmalbek Abdusalomov, Nodirbek Baratov, Alpamis Kutlimuratov, Taeg Keun Whangbo

**Affiliations:** 1 Department of IT Convergence Engineering, Gachon University, Sujeong-Gu, Seongnam-Si 461-701, Korea; akmaljon@gachon.ac.kr (A.A.); nod.baratov@gmail.com (N.B.); 2 Department of Information-Computer Technologies and Programming, Tashkent University of Information Technologies named after Muhammad Al-Khwarizmi, Tashkent 100200, Uzbekistan; alpamis92@gachon.ac.kr; 3 Department of Computer Engineering, Gachon University, Sujeong-Gu, Seongnam-Si 461-701, Korea

**Keywords:** fire detection, flame detection, YOLO networks, surveillance system, fire-like lights

## Abstract

Currently, sensor-based systems for fire detection are widely used worldwide. Further research has shown that camera-based fire detection systems achieve much better results than sensor-based methods. In this study, we present a method for real-time high-speed fire detection using deep learning. A new special convolutional neural network was developed to detect fire regions using the existing YOLOv3 algorithm. Due to the fact that our real-time fire detector cameras were built on a Banana Pi M3 board, we adapted the YOLOv3 network to the board level. Firstly, we tested the latest versions of YOLO algorithms to select the appropriate algorithm and used it in our study for fire detection. The default versions of the YOLO approach have very low accuracy after training and testing in fire detection cases. We selected the YOLOv3 network to improve and use it for the successful detection and warning of fire disasters. By modifying the algorithm, we recorded the results of a rapid and high-precision detection of fire, during both day and night, irrespective of the shape and size. Another advantage is that the algorithm is capable of detecting fires that are 1 m long and 0.3 m wide at a distance of 50 m. Experimental results showed that the proposed method successfully detected fire candidate areas and achieved a seamless classification performance compared to other conventional fire detection frameworks.

## 1. Introduction

One of the most common natural disasters worldwide affecting human life is fire, and the number of occurrences is increasing every year. Fires can cause loss of life or damage to property and typically cause significant economic damage in their wake. The primary causes of fires can be divided into two broad categories: natural and man-made fires. Dry climate, wind, smoking, heating appliances, chemical fires, and cooking conditions are ideal for fires to break out. These types of accidental fires can ignite with frightening unpredictability and spread uncontrollably in seconds. Early identification and prevention of the circumstances of these risks help to avoid unexpected fires and keep people safe. According to the statistics of the National Fire Agency of South Korea, the volume of property damage caused by fires surged to a record volume, and a total of 40,030 fires occurred in the nation in 2019, resulting in 284 deaths and 2219 injuries [1]. Therefore, fire and smoke detection methods have been introduced circumstantially by different research organizations.

Although several early warning and identification devices have been implemented to detect specific fire and flame properties over the last decades, such as fire alarm systems, sensor-based frameworks, and sensing technologies, several problems remain unresolved [2]. Recent research has shown that computer vision and deep-learning-based techniques have achieved great success and play a vital role in the field of fire detection. In particular, computer vision and artificial intelligence (AI)-based methods, such as static and dynamic texture analysis [3], convolutional neural networks (CNNs) [4], and 360-degree sensors [5], have been commonly applied to fire detection environments. 

To address the aforementioned problems, we present a robust, reliable, and automatic fire detection approach based on a new special CNN using the existing YOLOv3 framework. In this study, we focused on detecting an unforeseen fire to protect people’s lives and important properties. Fires have a wide range of sizes, colors, motions, shapes, speeds, appearances, or combinations of these features. Although these factors make fire detection challenging, we assume that there is still a great prospect to create such systems for automatic use.

The main contributions of the proposed method are as follows:

(1) We created a large dataset for the fire detection area with various scenarios of fire and flame (day and night), which will be made publicly available on the Internet. In a deep CNN, important features are learned using large databases to predict accurately and overcome overfitting problems.
(2) We propose a YOLOv3-based improved fire detection approach to increase the level of robustness and eliminate the time-consuming process.
(3) A method was developed to automatically move labeled bounded boxes when the fire dataset is turned to 15° every time.
(4) We used independent logistic classifiers and binary cross-entropy loss in YOLOv3 for class predictions during training. It has the advantage of being much faster than other detection networks with comparable performance.
(5) We reduced the number of false positives in the fire detection process by using fire-like images and removing low-resolution images from the dataset. In addition, it significantly decreased the average precision rate of inaccurately detecting small fire regions.

The remainder of the paper is organized as follows: Section 2 reviews existing conventional studies for the identification of specific fire properties. Section 3 presents the proposed fire detection approach in detail. The experimental results based on our databases are discussed in Section 4. Section 5 highlights certain limitations of the proposed method. Finally, Section 6 concludes the paper by summarizing our findings and future research directions.

## 2. Related Work

In general, existing systems related to automatic fire detection technologies can be divided into two categories: traditional fire detection approaches based on computer vision and AI-based fire detection systems using machine learning (ML) and deep learning (DL). In this section, we focus mainly on discussing the aforementioned two approaches. However, these features are not sufficient to efficiently and accurately detect fires. To overcome these limitations, additional information is required on geometric features, such as the location, shape, light source, and surface of the flame [6,7].

### 2.1. Computer Vision and Image Processing Approaches for Fire and Smoke Detection

Toulouse et al. [8] developed a new method focused on detecting the geometrical characteristics of flames, such as the position, rate of spread, length, and surface. They categorized the pixels of the fire image according to the color of the fire and the presence of smoke; however, non-refractory pixels were classified according to the average intensity of the corresponding image. Jian et al. [9] introduced an improved boundary detection operator, the Canny edge detector, which uses a multi-step operation. However, the aforementioned computer-vision-based frameworks have only been applied to simple and stable fire and flame images. Other researchers have used new algorithms based on FFT and wave variation to analyze the contours of forest fires in videos [10]. Earlier research has shown that these approaches are appropriate only under certain conditions.

Foreground and background images were analyzed to detect fire using color pixel statistics. For example, Turgay [11] developed a real-time fire detector that combines colored data with registered foreground and background frames. Color information about the fire is determined by statistical measurements of sample images containing fire. Simple adaptive background data scenes are employed using three Gaussian filters, each of which is utilized to model the pixel value color information in each color channel. However, color-based flame and fume recognition techniques are not possible because these strategies are not independent of environmental representatives, such as brightness, shadows, and other disturbances. Moreover, color-based methods are weaker than the dynamic changes in fire and smoke, although fire and smoke have long-term dynamic movements.

In [3], researchers implemented fire detection systems based on the analysis of the dynamic textures of smoke and flames using linear dynamic systems (LDSs). Their modeling, which combines color, movement, and spatial–temporal properties, has led to high levels of detection and a significant reduction in false alarms. To increase the effectiveness of the method employed employed to classify candidate areas for an early warning fire-detection tracking system using a two-class support vector machine classifier approach. An analysis of the temporal and spatial dynamic textures of the fire was performed to detect forest fires [12]. The dynamic texture properties were obtained using two-dimensional (2D) spatial wave fragmentation in the temporal field and three-dimensional (3D) volumetric wavelet fragmentation. In immobile texture investigation, hybrid surface descriptors were used to create an important feature vector to distinguish flames and distortions from traditional texture descriptors. One challenge of these methods is that they rely on data that are clearly visible in the detection of fires in image frames. The color, speed of movement, environment, size, and edges of the fire play an important role in deciding its occurrence. The quality of image and video files, weather conditions, and cloudy skies hinder the implementation of these techniques. Therefore, it is necessary to improve these methods using the latest supplementary approaches.

### 2.2. Deep Learning Approaches for Fire and Smoke Detection

In recent years, DL approaches have been significantly and effectively implemented in fire and smoke detection research areas in different ways. In contrast to the techniques reviewed earlier that rely on handcrafted characteristics, DL approaches can automatically select and remove complicated point descriptions. Another benefit is that deep neural networks can be implemented flexibly and successfully in automatic feature extraction using learned data; instead of spending time extracting functions, they can be modified to create a robust database and an appropriate network structure.

In our earlier work [4], we proposed a new fire detection method based on a DL approach, which uses a CNN that employs dilated convolutions. We evaluated our method by training and testing it on our custom-built dataset, which included the images of fire and smoke that we collected from the Internet and labeled manually. The proposed method is fully automatic, requires no manual intervention and was designed to be generalizable to unseen data. It offers effective generalization and reduces the number of false alarms. Based on the proposed fire detection method, our contributions include the following four main features: the use of dilation filters, a small number of layers, small kernel sizes, and a custom-built dataset, which was used in our experiments. This dataset is expected to be a useful asset for future research that requires images of fire and smoke.

Ba et al. [2] developed a new CNN model, SmokeNet, which incorporates spatial and channel-wise attention in CNN to enhance feature representations for scene classifications. Luo et al. [13] proposed a flame recognition algorithm based on the motion properties of smoke and a CNN. First, they distinguished the candidate pixels based on the background dynamic frame and foreground dynamic frame references. Subsequently, the highlights of the candidate pixels were automatically extracted by a CNN containing five convolutional layers and three fully connected layers. In [14], Park et al. proposed a fire detection method for an urban environment using static ELASTIC-YOLOv3 for the nighttime environment. As the first step of the algorithm, they proposed the use of ELASTIC-YOLOv3, which can improve the detection performance without increasing the number of parameters by improving YOLOv3, which is limited to the detection of small objects. In the second step, they proposed a method to generate a dynamic fire tube according to the characteristics of the flame. However, conventional nighttime fire flame detection algorithms face the following shortcomings: a loss of color information, relatively high brightness value compared to the surroundings, various changes in the shape and size from light blurring, and movements of the flames in all directions; in contrast, daytime flames tend to move in an upward direction. To analyze a fire emergency scene, a new approach was introduced recently to use deep convolutional image segmentation networks to identify and classify objects in a scene based on their build material and their vulnerability to catch fire [15].

In [16], a novel image fire detection algorithm based on the CNN models proposed in this study achieved an average precision accuracy of 83.7%. Furthermore, in [17,18,19,20], the CNN approach was applied to improve the performance of image fire detection technology. DL-based methods require significant training data, validation data, and test data. In addition, CNN has a problem with overfitting, and it is typically computationally expensive because it requires a large dataset for training. To address these problems, we created a large dataset, and the image datasets related to our study will be made publicly available.

## 3. Proposed Fire Detection Architecture

### 3.1. Dataset

One main limitation of fire detection is the inadequacy of the database to implement and analyze the proposed method. To address this problem, we attempted to use several computer vision techniques to increase the number of dataset images. Firstly, we collected fire images to build our dataset on which our model would be trained. The fire images used for the training tasks were collected from publicly available datasets and Google images. It is clear that the training dataset was limited in the case of fire detection. Then, we searched the Internet for fire videos with different conditions (size, shape, and color) and extracted (captured) them to the frames. Our training dataset comprised 9200 day and night fire images, as presented in Table 1. However, the expected result could not be achieved with this database, and we cannot expect good fire detection rates in real scenarios. Thus, we attempted to increase the number of fire frames and final accuracy using image augmentation techniques. The following subsection explains, in detail, the creation of our dataset.

**Figure 1:**
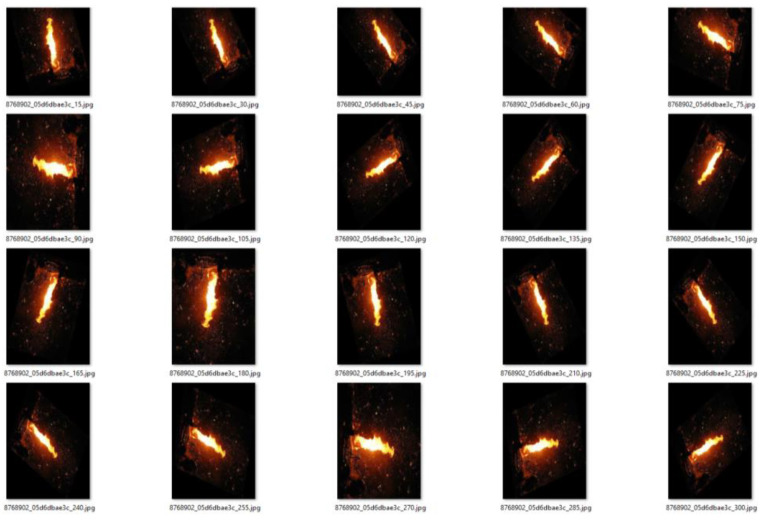
Examples of fire image rotation process.

We increased the number of images in the dataset by rotating each collected image at 15° angles from 360°, as shown in Figure 1. Dataset augmentation artificially expands the training set by creating modified copies of the examples already present. After applying this method, we acquired 23 times more images than before. Consequently, each image generated 23 augmented frames. As mentioned earlier, our dataset comprised 9200 images. After augmentation, the total number of datasets exceeded 211,600. In addition, more than 20,000 fire-like images were added to prevent false-positive results, as presented in Table 2. The effectiveness of CNN architectures significantly depends on the number of training datasets. Hence, it is important to extend the training dataset by data augmentation.

**Figure 2:**
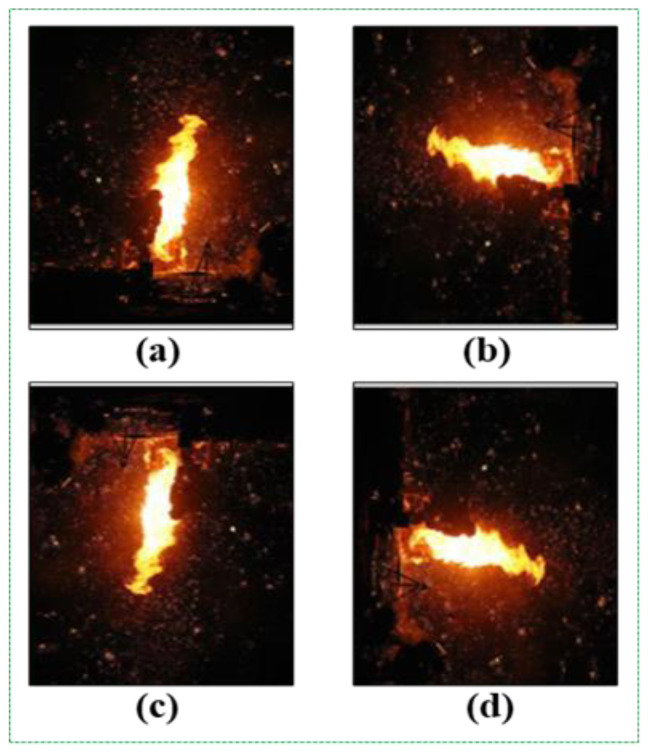
Original image (**a**); 90° rotation (**b**); 180° rotation (**c**); 270° rotation (**d**).

Firstly, we rotated all fire images to 90°, 180°, and 270° (Figure 2). When we rotated the fire images to a value greater than 15°, there was no significant change in the results obtained. Conversely, when we set the rotation degree above 15, we were likely to lose the region of interest (ROI) of the fire images.

Then, we used the LabelImg tool to appropriately label the fire in each image for YOLO training. The labeled file is a TXT file that stores the coordinates of the flame in the image. Moreover, it is employed on the CNN as part of the training process. Furthermore, we added fire-like images to the training set, but the label file contained empty TXT files. The purpose of adding these non-flammable images during training is to reduce the number of false-positive detections.

Rotating the labeled images at specified angles naturally changes the coordinates of the flame in the image. If we manually labelled it again, we would lose a considerable amount of time. Therefore, we should read all the pictures in the folder and turn them into corners and create a special program that changes their labels. Hence, we used the affine transformation method. Image transformation can be expressed in the form of the multiplication of the matrix by affine transformation, as detailed in [21].

### 3.2. System Overview

**Figure 3:**
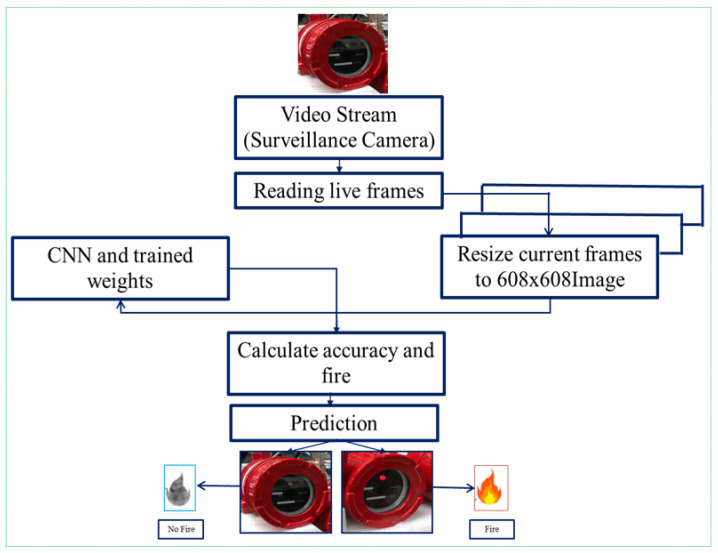
Overall process of the proposed method.

In this subsection, we provide a brief overview of the proposed method for detecting fire candidate areas quickly and accurately, irrespective of the size and shape of fires. In our approach, several techniques were developed to achieve our goal. As illustrated in Figure 3, we first captured live video sources from a real-time fixed camera. Secondly, we resized the input images to 608 × 608 pixels using the OpenCV framework. In our study, we also used 320 × 320 and 416 × 416-sized images, but they decreased the image size, resulting in a decrease in the flame detection accuracy and loss of important features of the fire images.

**Figure 4:**
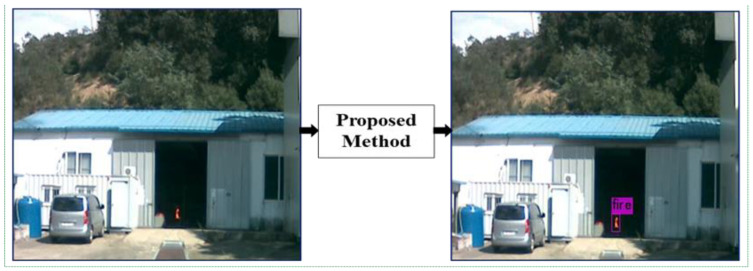
Fire detection process of the proposed method.

Before delivering the resized image to the CNN network, we employed data augmentation and image contrast enhancement approaches. Data augmentation was conducted to provide additional fire images for training the YOLOv3 network. Thirdly, we ran the network based on pretrained weights by initializing the model. Eventually, we evaluated the accuracy and predicted the occurrence of a fire. Object confidence and class predictions in YOLOv3 were predicted by logistic regression that used cross-entropy error terms for predicting fire scores. When fire zones were detected, a red light was illuminated in the camera and an emergency alarm signal was sounded. The suggested technique was employed for environmental monitoring and surveillance system applications, as shown in Figure 4. Our method successfully performed early-stage detection, even in very small fire regions.

### 3.3. Fire Detection Process

In recent years, the YOLO network has been used by several researchers to detect moving or static objects. However, there are several types of YOLO versions, not all of which may be effective in detecting fires. Therefore, we began our study by testing networks with the currently available 9200 fire images. YOLO is the fastest and most accurate real-time object detection algorithm that identifies specific objects in videos, live feeds, or images, such as cars (numbers), pedestrians, and animals. YOLO uses features learned by a deep CNN to detect an object. In this study, we used this algorithm for fire detection. Firstly, we tested the latest versions of YOLO algorithms, that is YOLOv3, YOLOv4, and their tiny versions, for fire identification cases to check the accuracy of the prediction of fire candidate regions/pixels with a limited dataset. The algorithms were evaluated using default versions without any changes in the training and testing process with 50,000 iterations, as presented in Table 3. One strength of the default path algorithms is that they have automatic color augmentation during training, which serves to increase accuracy during training. We used hue = 0.1, saturation = 1.5, and exposure = 1.5 as default.

**Figure 5:**
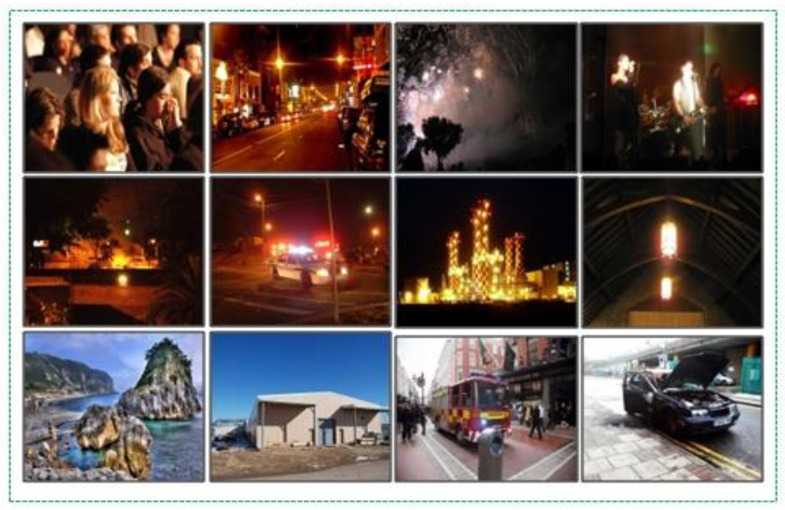
Fire-like lights.

For all algorithms, we set the input image size to 608 × 608 in the same manner. As presented in Table 3, the results were obtained in terms of the training and testing accuracy with different indicators. YOLOv3 scored the highest, with 82.4% accuracy in training and 77.8% accuracy in testing for 57 h. The next highest was YOLOv4, with 81.1% and 74.3% accuracy in training and testing, respectively, for 98 h. Although the YOLOv3 and YOLOv4 results were close to each other, the difference between the times they spend was large for a small weight size. It was technically expensive, and researchers might spend more time if they do not require devices to run DL methods. Another challenge of YOLO methods was that when we tested these trained weights files, most errors occurred in false positives. That is, it detected the fire even in non-fire pictures because fire-like scenarios (environments) were incorrectly classified as fires and warnings. Human eyes easily distinguish fire-like lights, but computers sometimes incorrectly classify neon signs, streetlights, and the headlights of vehicles as real fires because they have similar brightness, shape, and reflection (Figure 5). Thus, we selected YOLOv3 from the YOLO methods for our fire detection studies and decided to improve its results in fire detection cases.

**Figure 6:**
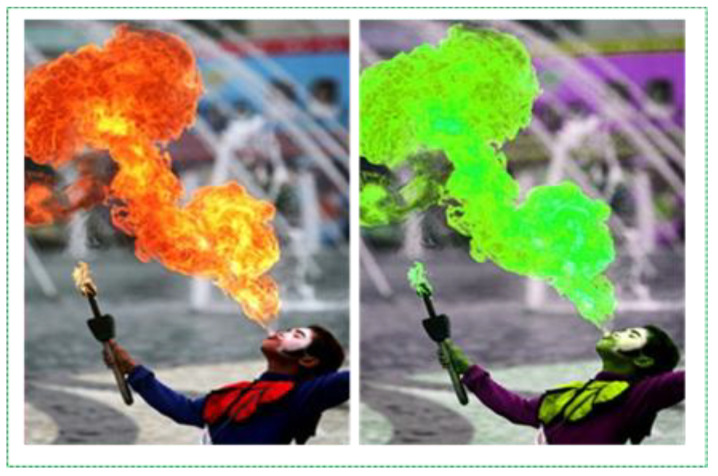
Fire images before and after hue augmentation.

This caused false alarms and increased the inconvenience in real time. After these errors, we made certain changes to the second experiment. After reviewing all the parameters in the training, we realized that color-based augmentation reduced the training accuracy instead of improving it. We realized that it was good that this augmentation was not used for our data because the fire did not have a specific shape and the importance of color retention was high. In Figure 6, we can observe the data changes when hue, saturation, and exposure are randomly used during training. The color of the flame changes to a completely different color, and training leads to an error.

We made certain changes to the dataset and algorithm to increase the accuracy. We deleted low-quality images from the dataset that were smaller than 608 × 608 pixels. In addition to the data, we multiplied the data that were not labeled, which caused errors. We decided not to automatically use the hue, saturation, and exposure parameters in the algorithms during training: hue = 0, saturation = 0, and exposure = 0. Moreover, before training the images, we increased the amount of data by changing the contrast and brightness through certain values. According to Szeliski [22], pixel transformations and local operators can be used to process images. In pixel transformations, the value of each output pixel depends only on the corresponding input pixel values. Brightness and contrast are good examples of pixel transformations.

*ց* (*x*) = *α*
*f*(*x*) + *β* (1)

In the above formula, α > 0 and *β* are commonly referred to as gain and bias parameters, respectively, and these parameters affect the contrast and brightness. *f*(*x*) denotes the source pixel of the image and *ց* (*x*) denotes the output pixel of the image. To simplify the aforementioned statement, the following equations can be used (2).

**Figure 7:**
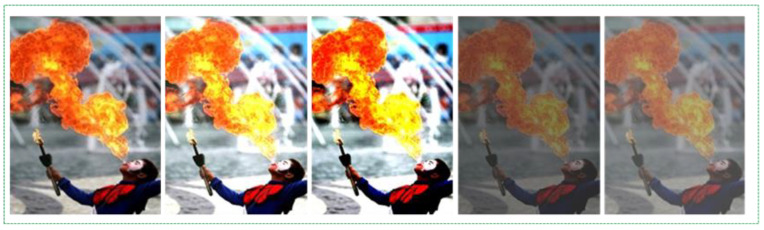
Fire images before and after hue augmentation.

*ց* (*i*,*j*) = *α*
*f*(*i*,*j*) + *β* (2)

where *i* and *j* indicate that the pixel is located in the *i*-th row and *j*-th column. By changing the values of *α* (contrast [1.0–3.0]) and *β* (brightness [0–100]), we created new augmented data in the dataset by changing the contrast and brightness, as shown in Figure 7.

As stated in Section 3.1, we had 211,600 fire images and 20,000 fire-like images. As aforementioned, after deleting low-quality and low-resolution images from the dataset, we obtained a total of 208,300 images. After changing the contrast and brightness of the fire images, we increased the total number of images to 624,900, as shown in Table 4. Firstly, we doubled the contrast of the original input images. Then, we reduced the brightness of the original image by half.

In the next part of our experiment, we tested only the YOLOv3 network with our final dataset using the same input size and iterations as in the first part. The results of our second experiment were significantly different from those of the first experiment. Our improved YOLOv3 network achieved seamless accuracy compared to other traditional YOLO networks, as summarized in Table 5.

Furthermore, we checked all YOLOv networks with the increased fire dataset (624,900) and compared the final accuracy. It can be noted from Table 6 that YOLOv3 ranked highest in training with 98.3% accuracy. YOLOv4 scored 96.1%, with a difference of 2.2% from YOLOv3 and barely lagged behind YOLOv3 in the test section. This was followed by YOLOv3 tiny_3l and YOLOv4 tiny_3l. Algorithms took more time than in previous experiments because of the increased number of dataset images.

**Figure 8:**
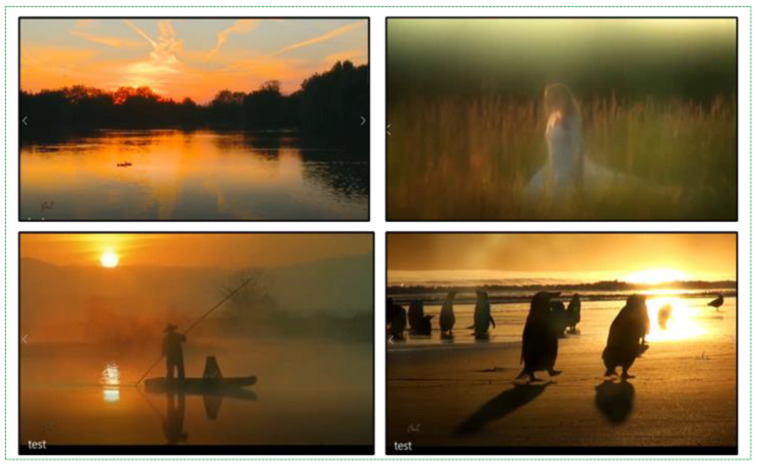
Example of sunlight images in the dataset.

After conducting all the training and testing experiments, we tested an additional 28,000 daytime pictures that were non-fire but similar to the fire pixels. These fire-like images have not yet been included in our dataset. As mentioned earlier, several errors in real-time fire detection occur in the case of a false alarm. The number of false positives out of these 28,000 images also helped to check the performance of the trained weights. Generally, sunlight distracts fire detection cameras, therefore, we should increase our dataset even more with images such as sunrise and sunset. Examples of daytime fire-like images are presented in Figure 8.

**Figure 9:**
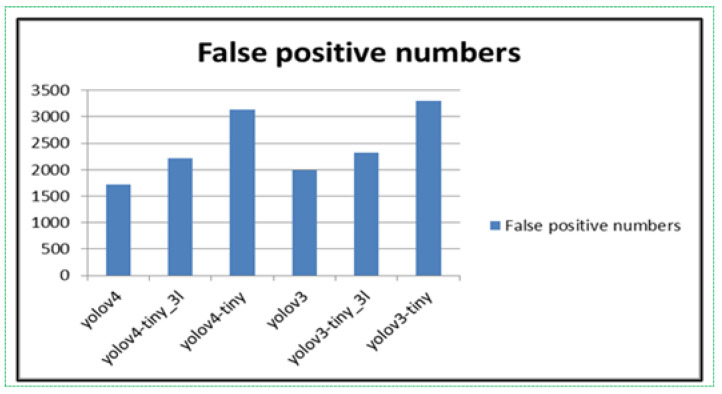
First experiment’s weighted file results on false-positive tests.

We tested the training weights from both experiments on a non-fire dataset. YOLO algorithms “score” regions based on their similarities to predefined classes. High-scoring regions are noted as positive detections of whatever class they most closely identify with. We can observe the results in Figure 9.

**Figure 10:**
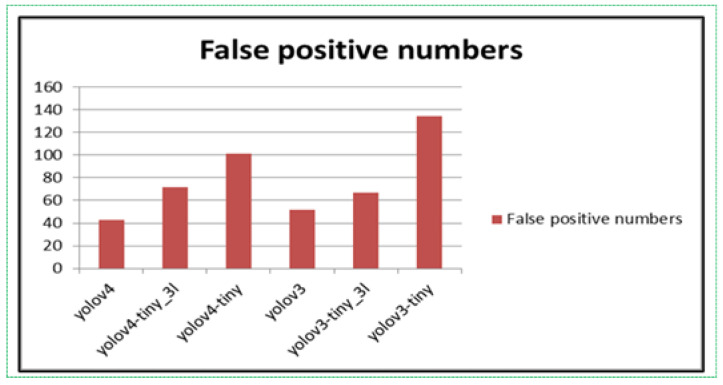
Second experiment’s weighted file results on false-positive tests.

The weighted files trained in the default algorithms recorded as many errors as shown in Figure 9. Even though the fewest errors were shown by the YOLOv3 and YOLOv4 algorithms, it was classified as having a large false-positive detection rate. After adding fire-like images to the dataset, the weighted files were trained again, and the experiments showed 20 times fewer errors than the weighted files in the default algorithms, as shown in Figure 10.

**Figure 11:**
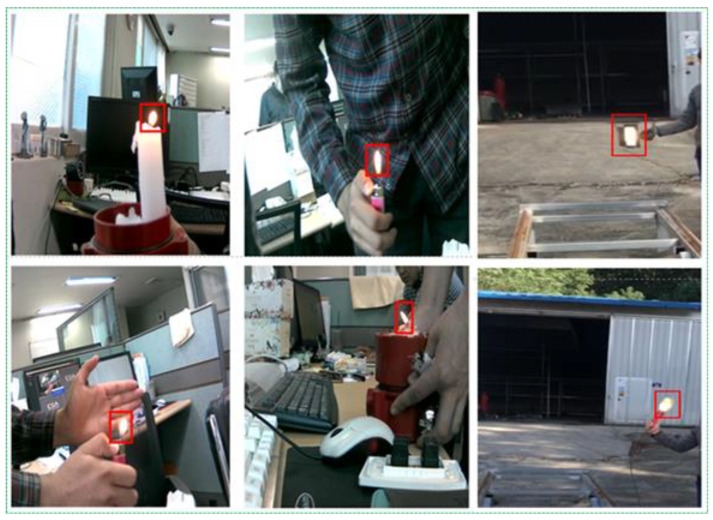
Small size fire region images for the training dataset.

While we achieved 98.3% accuracy in this step, we reviewed and analyzed several recently introduced approaches to improve this result. We observed from [23] that detecting small-sized fire images is not an easy task, and most methods fail to detect them. To overcome this challenge, we created small-sized fire images to increase our dataset and improve our final accuracy, as depicted in Figure 11. We employed a large-scale feature map to detect small moving objects and concatenate them with a feature map from earlier layers, which helps preserve the fine-grained feature, as mentioned in [16]. This large-scale feature map with the location information of the previous layers and complex features of deeper layers was applied to identify small-sized fire pixels.

**Figure 12:**
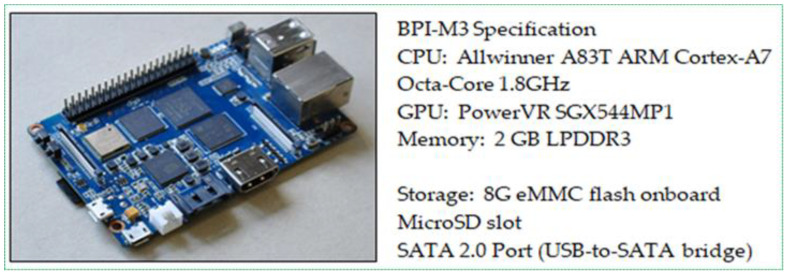
Characteristics of Banana Pi M3.

We improved the fire detection accuracy to 99.7%. With this result, we can detect any type of fire in the early stage, even on a very small scale. Finally, we adopted our method on the Banana Pi M3 (BPI M3) board, as shown in Figure 12, following which, the proposed method can be used with a smaller CNN to achieve a reduced processing time without any loss in accuracy. Running on BPI M3, a large CNN causes it to run very slowly and is technically expensive. To perform this task, we used only three layers of the improved YOLOv3. In the next section, we compare our method with existing methods to analyze the efficiency and performance.

## 4. Experimental Results and Discussion

**Figure 13:**
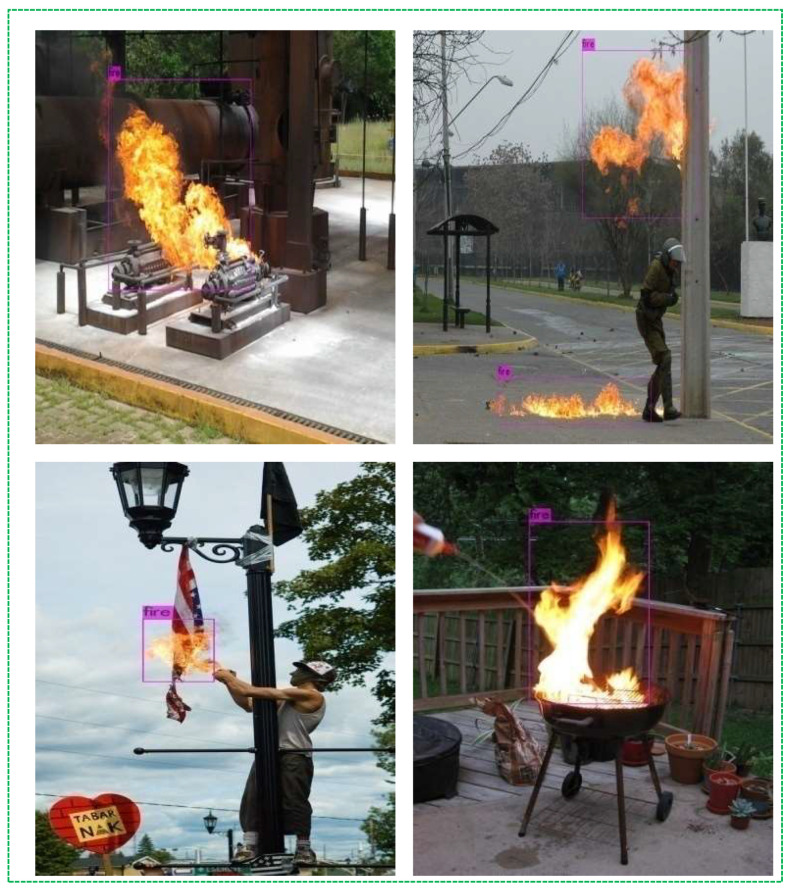
Visible experiments in different environments.

We implemented and tested the proposed method in Visual Studio 2015 C++ on a PC with a 3.20GHz CPU, 32GB RAM, and two Nvidia GeForce 1080Ti GPUs. To evaluate the performance of the fire detection method, the system was tested in different environments (mountain, industry, and social life). The previous section discussed several experiments that were conducted and implemented using the YOLOv models. However, in this section, we discuss the strengths and limitations of traditional fire detection methods and our method. Figure 13 illustrates the examples of certain visible experiments in outdoor environments using our improved YOLOv3 and the Darknet-53 classifier [24]. Darknet-53 has 53 convolutional layers, which makes it a more powerful and efficient feature extractor than previous versions. Experimental results indicated that our improved fire detection method accurately detected fire spread; in contrast, several methods failed and misclassified them as moving objects. Certain moving objects had the same color intensities and similar movements as fire pixels in the background area. In addition, our method worked effectively, even when there were several fires in the frame sequences.

In this section, we discuss the demonstration of a quantitative analysis to compare the performance of different strategies. We compared our method with well-known fire detection algorithms, which are based on YOLO networks and DL approaches. We used the results in their papers for comparison, but we are not sure whether they are true because source codes and datasets of these methods are not publicly available to check the real performance. We computed metrics such as F-measure (FM), precision, and recall, as in our earlier study [4]. The FM score is the weighted average that balances measurements between the means of precision and recall rates. Hence, this score considers both false positives and false negatives. Intuitively, it is not easy to understand accuracy, but FM is more common than accuracy. Accuracy works best if false positives and false negatives have similar costs. If the costs of false positives and false negatives are different, it is better to consider both precision and recall. Precision is the ratio of correctly predicted positive observations to the total predicted positive observations. Recall is the ratio of correctly predicted positive observations to all observations in the actual class, as expressed in Equation (3). The proposed method’s average of FM, recall, and precision was 98.9%. False detection occurred in 1.1% of cases, owing to the blurring of objects at night. To calculate the average precision and recall rates of the shadow remover methods, the following equations can be used:

(3) $$\begin{array}{c} Precision=\frac{TP}{TP+FP}\\ Recall=\frac{TP}{TP+FN} \end{array}$$

where *TP* denotes the number of true positives indicating correctly detected fire regions, *FP* denotes the number of false positives, and *FN* denotes the number of false negatives. *Precision* is defined as the number of true positives over the number of true positives and the number of false positives. *Recall* is defined as the number of true positives over the number of true positives and the number of false negatives.

Then, F-Measure, indicated as *FM*, is calculated using (4), considering both *precision* and *recall*.

(4) $$FM=\frac{2\times precision\times recall}{precision+recall}$$

In actual applications, fire regions/pixels in natural scene images may be extremely dark, blurred, or blocked (by cloud). In addition, we used the Jaccard index to assess the performance of the fire detection methods. The Jaccard index, also known as the intersection-over-union (*IoU*) metric, expresses the number of objects two sets have in common as a percentage of the number of objects they have in total. In other words, it is an effective metric for evaluating detection results and is defined as the area of overlap between the detected fire region and the ground truth divided by the area of union between the detected fire region and the ground truth (5):

(5) $$IoU=\frac{groundTruth\cap prediction}{groundTruth\cup prediction}$$

*FM* score and *IoU* values range between 0 and 1, where these metric scores reach their best values at 1. The evaluation of our method and other recently published fire detection methods is presented in Table 7.

In Table 8, we summarize the results of the performance of the methods used in fire detection based on quantitative and qualitative experiments. Based on the results of the analyzed methods, we evaluated the scores for different properties. Our proposed approach does not suffer from unwanted and unnecessary noise of pixels, and it does not depend on the fire direction, number of objects, and types of scenes (sunny or cloudy days). In a normal environment, the best results were obtained for solving early fire detection problems using the proposed method with reduced processing time.

The results of fire detection methods are classified as powerful, normal, and not strong for the seven categories. Powerful criteria indicate that the algorithm can be implemented for all types of events. Normal criteria indicate that the algorithm may fail in certain cases, such as when it occurs strongly or spreads the fire. Not strong criterion indicates that algorithms are not reliable for noise or color, and many times distort the original shape of moving fires during the fire detection process. Fire spread detection indicates that fire detectors successfully detected multiple direction shadows from images.

Furthermore, we compared the calculation processing time per frame (fps) with the different resolutions of the input layer on the BPI M3 platform, which is widely used in object detection and other fire detection studies. Higher resolution images for the same model have better prediction accuracy but are slower to process. In our research, we used the RGB (red, green, blue) color model because it has less computational complexity than other color models and, hence, is adopted to describe fire pixels. We found out that, if the non-fire images and fire images are not distinguished for detection, the time complexity of the method will be greatly increased. Table 9 shows the comparison results.

## 5. Limitations

**Figure 14:**
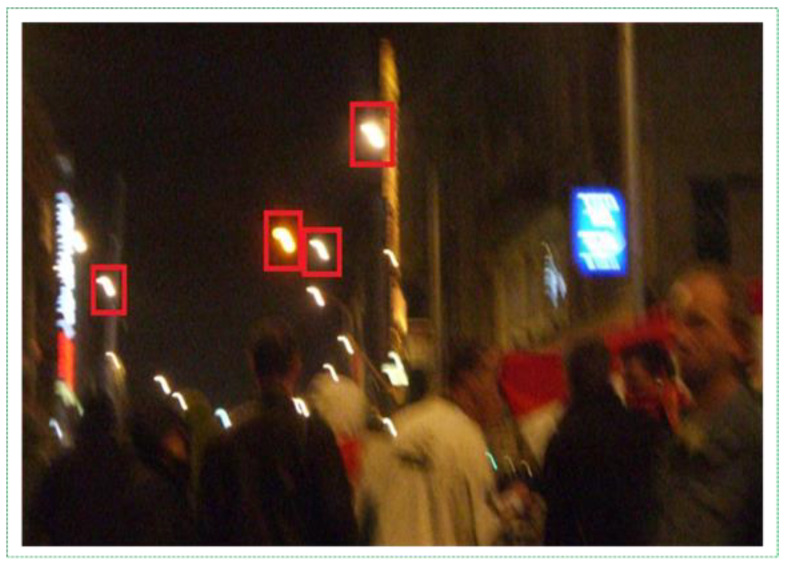
Blurred lamps at night time environments.

It is difficult to say that the methods proposed thus far do not have any shortcomings. Our proposed method may also cause errors and consider electrical lamps as real fires. This mainly occurs at nighttime because several objects suffer from blurring problems, as illustrated in Figure 14. Fire detection can be difficult during a rainy night. To overcome this problem, we are currently experimenting with datasets containing fire-like images at night in urban areas. In addition, in [33], a CNN-based model was proposed to solve blurry environments and generate sharp video frame sequences efficiently. We will apply this method to illuminate blurring aspects in the future. Irrespective of the aforementioned problems, the experimental results showed that our method is very robust and effective for fire detection (accuracy 99.7% and average FM 98.9%).

## 6. Conclusions

This paper presented a new approach for detecting and classifying the fire regions of surveillance fields based on YOLOv3. We first tested the default YOLO networks, that is YOLOv3, YOLOv4, and their tiny versions, without any modifications to select the best to use in our study. They are currently the fastest and most accurate object detection algorithms based on DL algorithms, although they are not widely used in the field of fire detection. We selected and used YOLOv3 to address and achieve the expected results irrespective of the size and color of the fire problems. We employed several techniques to improve the accuracy of YOLOv3 and achieve a high precision rate to detect fire candidate areas. The experiments showed that changing the algorithm (model size) and the dataset could quickly detect a real-time fire with a high degree of accuracy. Another advantage of our results is that false alarms were minimized. In addition, we adopted the proposed method on the BPI M3 board and provided the opportunity to run both CPU and GPU frameworks with reduced processing time compared to traditional fire detection approaches. We conducted experiments using our datasets to evaluate the effectiveness of our approach. The results demonstrated that the proposed method could easily ensure real-time fire safety in indoor and outdoor environments.

Future work will include improving the accuracy of our method and handling blurring issues in nighttime environments. Our future projection is to build a lightweight model with robust detection performance that would allow us to set up embedded devices with low computational capabilities.

## Figures and Tables

**Table 1:** Distribution of fire images in the dataset.

Dataset	Open Source Datasets	Video Frames	Total
Fire Images	4336	4864	9200

**Table 2:** Distribution of fire and fire-like images in the dataset.

Dataset	Training Images	Testing Images	Total
Fire Images Fire-like Images	205,717 20,000	5883 0	211,600 20,000

**Table 3:** Making pretrained weights using a limited dataset.

Algorithm	Input Size	Training Accuracy (ap50)	Testing Accuracy (ap50)	Weight Size	Iteration Number	Training Time
YOLOv4	608 × 608	81.1%	74.3%	245 MB	50,000	98 h
YOLOv4-tiny_3l		77.8%	71.8%	23 MB		22 h
YOLOv4-tiny		69.02%	62.9%	23 MB		21 h
YOLOv3		82.4%	77.8%	236 MB		57 h
YOLOv3-tiny_3l		75.6%	72.4%	33.7 MB		26.5 h
YOLOv3-tiny		70.9%	64.2%	33.7 MB		22 h

**Table 4:** Distribution of all fire images in the dataset.

Before	After Filtering	After Contrast Increase (Double)	After Contrast Decrease (Half)
211,600	208,300	208,300	208,300

**Table 5:** Making pretrained weights using YOLOv3.

Algorithms	Input Size	Training Accuracy (ap50)	Testing Accuracy (ap50)	Weight Size	Iteration Number	Training Time
YOLOv4	608 × 608	81.1%	74.3%	245 MB	50,000	98 h
YOLOv4-tiny_3l		77.8%	71.8%	23 MB		22 h
YOLOv4-tiny		69.02%	62.9%	23 MB		21 h
YOLOv3		98.3%	97.8%	236 MB		85 h
YOLOv3-tiny_3l		75.6%	72.4%	33.7 MB		26.5 h
YOLOv3-tiny		70.9%	64.2%	33.7 MB		22 h

**Table 6:** Comparing all YOLO networks based on a large dataset.

Algorithms	Input Size	Training Accuracy (ap50)	Testing Accuracy (ap50)	Weight Size	Iteration Number	Training Time
YOLOv4	608 × 608	96.1%	95.3%	245 MB	50,000	103 h
YOLOv4-tiny_3l		94.2%	89.9%	23 MB		37 h
YOLOv4-tiny		88.3%	85.1%	23 MB		33 h
YOLOv3		98.3%	97.8%	236 MB		85 h
YOLOv3-tiny_3l		95.6%	91.4%	33.7 MB		39 h
YOLOv3-tiny		85.3%	82.7%	33.7 MB		37.5 h

**Table 7:** Quantitative results of fire detection.

Algorithms	*P *(*%*)	*R *(*%*)	*FM *(*%*)	*IoU *(*%*)	*Average *(*%*)
ELASTIC-YOLOv3 [14]	98.5	96.9	97.7	96.9	**97.7**
YOLOv3-incremental [25]	97.9	91.2	94.3	93.8	**94.4**
Faster R-CNN [26]	81.7	94.5	87.2	89.2	**88.2**
Dilated CNNs [4]	98.9	97.4	98.2	98.7	**98.1**
AlexNet [27]	73.3	61.3	75.1	85.2	**79.9**
ResNet [28]	94.8	93.6	94.2	95.8	**94.3**
VGG16 [29]	97.5	87.9	92.7	91.9	**92.6**
YOLOv5 [30]	98.5	96.7	98.0	97.1	**97.9**
YOLOv3+OHEM [31]	86.6	77.8	89.2	86.3	**84.5**
YOLOv4 [32]	95.9	96.7	98.3	97.1	**96.9**
**Our Method (Improved YOLOv3)**	98.1	99.2	99.5	98.7	**98.9**

**Table 8:** Fire detection performance review using various features.

Criterions	YOLOv3 + OHEM [31]	Dilated CNNs [4]	ELASTIC–YOLOv3 [14]	Our Method (Improved YOLOv3)
**Scene Independence**	normal	powerful	normal	powerful
**Object Independence**	normal	powerful	powerful	normal
**Fire Independence**	not strong	powerful	normal	powerful
**Robust to Color**	normal	normal	not strong	powerful
**Robust to Noise**	powerful	normal	powerful	powerful
**Fire Spread Detection**	normal	not strong	not strong	powerful
**Computational Load**	not strong	powerful	normal	powerful

**Table 9:** Average frame processing time (in seconds) per sequence for various image sizes.

Input Resolution	Number of Frames (fps)	Processing Time (s)
608 × 608	1	0.26
416 × 416	1	0.24
320 × 320	1	0.23

## Data Availability

Not applicable.
